# Early findings from periscope (Pan-European response to the impacts of COVID-19 and future pandemics and epidemics)

**DOI:** 10.1192/j.eurpsy.2021.118

**Published:** 2021-08-13

**Authors:** W. Osika, C. Dalman, M. Niemi, P. Flodin, A. Sörberg Wallin, M. Asper, A.-C. Hollander, E. Pöllänen, O. Simonsson, K. Sörman

**Affiliations:** 1 Clinical Neuroscience, Karolinska Institutet, Stockholm, Sweden; 2 Department Of Global Public Health, Karolinska Institutet, Stockholm, Sweden

## Abstract

**Abstract Body:**

The H2020/PERISCOPE project, including 32 partners from European universities & agencies, began 1st November 2020 and will last 36 months. The overarching objectives of PERISCOPE are to map and analyse the unintended impacts of the COVID-19 outbreak; develop solutions and guidance for policymakers and health authorities on how to mitigate the impact of the outbreak; enhance Europe’s preparedness for future similar events; and reflect on the future multi-level governance in the health as well as other domains affected by the outbreak. During this session we will report about early lessons learnt from the mapping and assessments of the impacts of the COVID-19 outbreak on mental health at national and subnational level in the EU with respect to individuals, communities and societies. Further, we will comment on their comparability. The aim is to explore differences between countries regarding the occurrence of mental ill health, and especially the impact on vulnerable groups, and how this is related to exposure to SARS-CoV-2, differences in policies over time, and effects on the economy. We will reflect on the short- and long-term consequences on mental health and health inequalities, report on the ongoing development of holistic policy guidelines for health authorities & other authorities, and from the analysis of multilevel governance, at local, regional and national level, memberstate – EU-level, and EU - global governance level. PERISCOPE will continue collecting data and updating a common data ”Atlas”, which would lead the consortium to engage in modelling and experiments to provide “continuous nowcasting” of the outbreak.

**Disclosure:**

No significant relationships.

